# Supplementary material for: Blood–Brain Barrier Biomarkers before and after Kidney Transplantation
Source: Int J Mol Sci. 2023 Apr 1;24(7):6628. doi: 10.3390/ijms24076628 (PMC10095132; doi:10.3390/ijms24076628)
Supplement: Supplementary file 1 [file ijms-24-06628-s001.zip › ijms-2318027-supplementary.pdf]

SUPPLEMENT

Supplement Table S1. Clinical profile of male and female kidney transplant patients at baseline

Parameters	Males (n=52)	Females (n=22)	*p* value
Age (yrs)	46 (32-51)	46 (30-57)	0.9135
BMI (kg/m^2)	25 (23-27)	23 (21-24), n=21	0.0091*
SBP (mmHg	146 (130-163)	127 (121-151), n=21	0.0077*
DBP (mmHg)	89 (79-95)	80 (75-87), n=21	0.0159*
Vintage years	1.2 (0.6-3.3), n=25	1.1 (0.8-2.7), n=14	0.9827
Comorbidities			
CVD	44 (85)	18 (82)	0.7418
DM	4 (8)	2 (9)	>0.9999
Medications			
Ca Channel Blockers	33 (63)	7 (32)	0.0207*
Beta Blockers	36 (69)	12 (55)	0.2889
ACEi/ARBs	32 (62)	10 (24)	0.3046
Statin	15 (29)	5 (23)	0.7759
Biochemistry			
Creatinine, µmol/L	743 (613-926.50)	608.5 (523.8-687)	0.0020*
Albumin, g/L	36 (33-39), n=51	35.5 (32.8-37.0)	0.5879
hsCRP, mg/L	0.7 (0.4-2.0)	0.5 (0.3-1.6)	0.3355
Calcium, mmol/L	2.2 (2.1-2.4), n=51	2.3(2.2-2.4)	0.4112
Phosphate, mmol/L	1.6 (1.2-1.8), n=51	1.8 (1.4-1.9)	0.5117
Troponin T, µg/L	21 (14-39), n=51	21 (11.3-30.5), n=20	0.2351
Triglycerides, mmol/L	1.4 (1.0-1.9)	1.2 (0.9-1.7)	0.0655
Cholesterol, mmol/L	4.4 (3.63-5.0)	4.7 (3.9-5.6)	0.3570
HDL, mmol/L	1.3 (1.0-1.5)	1.6 (1.2-1.9)	0.0218*
Apo-A1, g/L	1.3 (1.1-1.5)	1.6 (1.4-1.7)	0.0441*
Apo-B, g/L	0.9 (0.7-1.0)	0.9 (0.8-1.0)	0.9368
Lp(a), mg/L	50.5 (12.3-12), n=36	79 (16-140), n=15	0.4947
HBA1c, mmol/mol	33 (30-37), n=51	36.50 (33.0-39.3)	0.0578
Homocysteine, µmol/L	36.5 (30.8-55.0), n=50	34 (25.3-43.3)	0.0874
Glucose, mmol/L	5.70 (5.3-6.2), n=21	5.2 (4.5-7.8), n=12	0.3494
25-OH-Vit.D, nmol/L	39 (29.3-63.5)	35 (22.5-56.8)	0.3361

Data are presented as median and interquartile range (Q1-Q3). The differences between baseline and two years post KT were analyzed using Mann-Whitney U test. Categorical data analyzed by Fisher's test. Significance was determined at $^*p < 0.05$
Abbreviations: BMI – body mass index; SBP; systolic blood pressure; DBP- diastolic blood pressure; CVD – cardiovascular disease; DM – diabetes mellitus; Ca- calcium; ACEi – angiotensin converting enzyme inhibitor; ARB – angiotensin receptor blocker; hsCRP – high sensitivity C-reactive protein; HDL – high density lipoprotein; Apo-A1 – apolipoprotein -A1; Apo-B – apolipoprotein – B; Lp(a) – lipoprotein(a); HBA1c – hemoglobin A1c.
